# FGF2, an Immunomodulatory Factor in Asthma and Chronic Obstructive Pulmonary Disease (COPD)

**DOI:** 10.3389/fcell.2020.00223

**Published:** 2020-04-02

**Authors:** Yuanyang Tan, Yongkang Qiao, Zhuanggui Chen, Jing Liu, Yanrong Guo, Thai Tran, Kai Sen Tan, De-Yun Wang, Yan Yan

**Affiliations:** ^1^Guangdong Provincial Key Laboratory of Biomedical Imaging and Guangdong Provincial Engineering Research Center of Molecular Imaging, The Fifth Affiliated Hospital, Sun Yat-sen University, Zhuhai, China; ^2^BGI-Shenzhen, Shenzhen, China; ^3^Department of Pediatrics, The Third Affiliated Hospital of Sun Yat-sen University, Guangzhou, China; ^4^Department of Respiratory Medicine, The Fifth Affiliated Hospital, Sun Yat-sen University, Zhuhai, China; ^5^Department of Physiology, Yong Loo Lin School of Medicine, National University of Singapore, Singapore, Singapore; ^6^Department of Otolaryngology, Yong Loo Lin School of Medicine, University Health System, National University of Singapore, Singapore, Singapore; ^7^Center for Interventional Medicine, The Fifth Affiliated Hospital, Sun Yat-sen University, Zhuhai, China

**Keywords:** asthma, chronic obstructive pulmonary disease, fibroblast growth factor 2, immunomodulation therapeutics, airway structural cells

## Abstract

The fibroblast growth factor 2 (FGF2) is a potent mitogenic factor belonging to the FGF family. It plays a role in airway remodeling associated with chronic inflammatory airway diseases, including asthma and chronic obstructive pulmonary disease (COPD). Recently, research interest has been raised in the immunomodulatory function of FGF2 in asthma and COPD, through its involvement in not only the regulation of inflammatory cells but also its participation as a mediator between immune cells and airway structural cells. Herein, this review provides the current knowledge on the biology of FGF2, its expression pattern in asthma and COPD patients, and its role as an immunomodulatory factor. The potential that FGF2 is involved in regulating inflammation indicates that FGF2 could be a therapeutic target for chronic inflammatory diseases.

## Introduction

Chronic inflammatory airways disease is a major economic burden and public health challenge worldwide. Asthma and chronic obstructive pulmonary disease (COPD) are the most prevalent among them, with 235 million people suffering from asthma (Whalen and Massidda, 2015) and 250 million from COPD (Al-Haddad et al., 2014). Though differing in etiology, asthma, and COPD share pathological features, which include chronic inflammation, airflow limitation, and airway wall remodeling (Holgate et al., 2015; Barnes, 2018). Chronic airway inflammation is central to disease pathophysiology, contributing to airway dysfunction and remodeling through the release of inflammatory mediators and interaction with airway structural cells, and therefore, is the primary therapeutic target (Holgate, 2012; Barnes, 2016). For a long time, anti-inflammatory drugs have been applied as first-line treatments for chronic airway diseases. Nevertheless, evidence suggests that the current drugs are not always effective in inhibiting chronic airway inflammation. For example, some asthmatic patients who respond poorly to corticosteroids also do not respond to anti-IL5/anti-IL13 treatments, leaving severe asthma a disease that has no effective cure (Astrazeneca, 2017; Drick et al., 2018). As for COPD patients, corticosteroids are inefficient partly because cigarette smoking impairs histone deacetylase 2 activity resulting in a lack of suppression of anti-inflammatory effects of corticosteroids (Barnes, 2004; Barnes et al., 2004). Moreover, anti-IL-5 treatments failed to show efficacy in severe COPD patients, and treatment with kinase inhibitors could impair innate immunity through inhibiting cellular components of inflammation, which evoke concerns about their usage (Gross and Barnes, 2017; Narendra and Hanania, 2019). In these situations, alternative treatments for chronic airway diseases, particularly asthma and COPD, are urgently needed. Recently, the immunomodulatory function of airway structural cells, e.g., airway epithelial cells (AECs), airway smooth muscle cells (ASMCs), and endothelial cells (ECs), has been attracting much interest. A myriad of articles showed that airway structural cells, regarded as immunomodulatory cells, are the targets for, and source of, various inflammatory mediators, in the perpetuation of the chronic inflammation via autocrine or paracrine pathways (Hahn et al., 2006; Tliba and Amrani, 2008; Al-Soudi et al., 2017). For instance, in the presence of inflammatory mediators, airway structural cells express adhesion molecules that are essential to the recruitment of inflammatory cells. This would trigger the production and secretion of multiple cytokines, chemokines, and growth factors, which in turn contributes to airway inflammatory states. Of note, the complex network between inflammation and airway structural cells has been rarely targeted by the current drugs and thus may open new avenues for potential anti-inflammatory therapies.

Fibroblast growth factor 2 (FGF2, also known as basic fibroblast growth factor, bFGF), a potent mitogen for fibroblasts, has been reported to be overexpressed in severe asthma and COPD, particularly patients with exacerbated COPD and smokers with chronic bronchitis (Redington et al., 2001; Kranenburg et al., 2005; Guddo et al., 2006; Pavlisa et al., 2010). Moreover, increased sputum FGF2 was reported to be a biomarker of airway remodeling and associated with lung function (Bissonnette et al., 2014). It is also evident that FGF2 plays a critical role in cell proliferation, differentiation, migration, and apoptosis in airway structural cells (Bosse et al., 2006; Yi et al., 2006; Zhang et al., 2012; Schuliga et al., 2013), contributing to epithelial repair, ASMCs hyperplasia, and vascular remodeling. Interestingly, some studies suggest that FGF2 may be associated with airway inflammation, and is a potential target for inflammatory diseases (Jeon et al., 2007; Kim et al., 2018; Laddha and Kulkarni, 2019). In this review, we will provide an overview of the immunomodulatory function of FGF2, particularly through airway structural cells-driven processes, identifying raising questions, and proposing future research directions for targeting FGF2 in asthma and COPD.

## FGF2 Discovery and Signaling

### Discovery of FGF2 and Its Isoforms

FGF was first discovered in pituitary extracts as a potent mitogen for fibroblasts in 1973 (Armelin, 1973). Then, FGF2 was isolated at basic pH and named “basic fibroblast growth factor,” with FGF1 (also known as an acidic fibroblast growth factor, aFGF) purified with acidic extraction condition. Since then, 22 FGF family members have been identified, with diverse physiological functions. FGF2 is encoded by the *FGF2* gene, a 3-exon gene localized on human chromosome 4q26–27 (Ornitz and Itoh, 2001). It has low (18-kDa) and high (22-, 22.5-, 24-, and 34-kDa) molecular weight isoforms, which are translated from a single transcript by starting from alternative, in-frame start codons. These isoforms have different expression patterns. Generally, the low molecular weight (LMW) FGF2 is considered cytoplasmic or/and nuclear and can be secreted. Of note, unlike most of FGF family members, LMW FGF2 lacks a classical amino-terminal signal peptide that directs secretion (Mignatti et al., 1992). However, it can be found anchored to extracellular matrix (ECM) components at the extracellular surface of the plasmalemma and within the basement membrane of different tissues (Folkman et al., 1988; Shute et al., 2004). More recent evidence suggests that LMW FGF2 can be released not only from damaged cells but also via an unconventional secretory pathway that is based upon direct protein translocation across plasma membranes as opposed to the traditional endoplasmic reticulum/Golgi apparatus-dependent protein secretion pathway (La Venuta et al., 2015). By contrast, The HMW FGF2 has been identified in the nucleus, with its additional amino-terminal sequences providing the nucleus-localization signal. Whilst several studies have identified that HMW FGF2 signaling is FGF receptor (FGFR)-independent, and the physiological function of HMW FGF2 remains unclear. Therefore, in this review, we will focus on LMW FGF2 (identified as FGF2, unless stated otherwise), for which FGF2 usually signal either in the cytoplasm without secretion or via representative membrane receptor activation to modulate subsequent downstream signaling events in an autocrine or paracrine pattern.

### FGF2 Signaling and Basic Function

**FIGURE 1:**
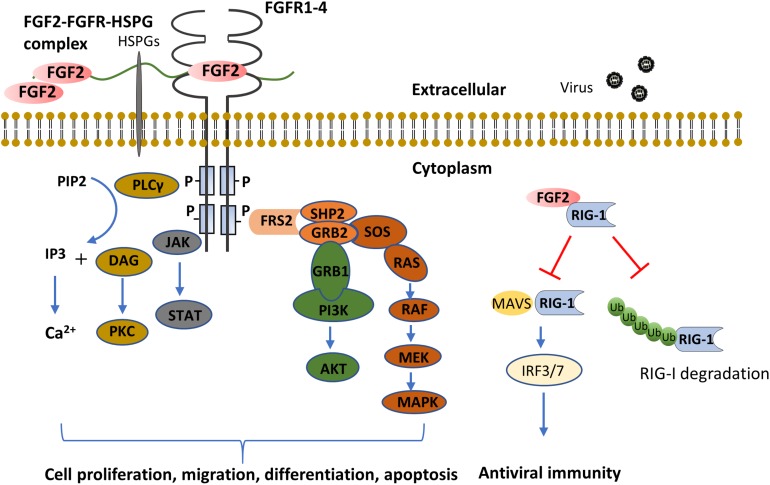
FGF2 functions through FGFR dependent or independent pathways. The binding of FGF2 to FGFR induces the formation of FGF2-FGFR-HSPG complex, which leads to receptor dimerization and transphosphorylation of tyrosine kinase domains. The major FGFR kinase substrate, FRS2α, is phosphorylated by the activated FGFR kinase and recruits the adaptor protein, GRB2 and SHP2. This results in subsequent activation of MAPK and PI3K-AKT pathways. In addition, the combination of FGF2 and FGFR also activates JAK and PLCγ, the former activates the STAT pathway, the latter hydrolyzes PIP2 into IP3 and DAG, and activates Ca^2+^ and PKC signaling, respectively. As compared to extracellular FGF2 signaling, cytosol FGF2 binds to RIG-1 to prevent RIG-1 degradation. While in viral infection, the binding of FGF2 with RIG-1 tends to prevent the binding of MAVS with RIG-1, and thus inhibit anti-viral innate immunity. (AKT, protein kinase B, also known as PKB; DAG, diacylglycerol; FGF2, fibroblast growth factor; FGFR, fibroblast growth factor receptor; FRS2α, FGF receptor substrate 2α; GRB1, growth factor receptor-bound protein 1; GRB2, growth factor receptor-bound protein 2; HSPG, heparan sulfate proteoglycan; IP3, inositol trisphosphate; IRF, interferon regulatory transcription factor; JAK, Janus kinase; MAPK, mitogen-activated protein kinase; MAVS, mitochondrial antiviral-signaling protein; MEK, mitogen-activated protein/extracellular signal-regulated kinase kinase; PI3K, phosphoinositide 3-kinase; PIP2, phosphatidylinositol (4,5)-bisphosphate; PKC, protein kinase C; PLC, phosphoinositide phospholipase C; RIG-1, retinoic acid-inducible gene 1; SHP2, src homology 2-containing phosphotyrosine phosphatase; SOS, son of sevenless; STAT, signal transducer and activator of transcription). (Klint et al., 1999; Maher, 1999; Sahni et al., 2001; Deo et al., 2002; Sulpice et al., 2002; Fernandes et al., 2004; Lin et al., 2011; Sufen et al., 2011; Willems-Widyastuti et al., 2013; Liu et al., 2015; Dolivo et al., 2017).

Four high-affinity receptor tyrosine kinases have been identified as FGFs receptors, comprising FGFR1 through FGFR4. Of note, FGFR5, recently discovered to interact with FGFs, has been proposed to act as a negative regulator of FGFs signaling in the light of lacking the tyrosine kinase domain (Sleeman et al., 2001). Once binding with FGFs, FGFRs undergo conformational changes leading to tyrosine kinase activation and subsequent the activation of intracellular signalings including mitogen-activated protein kinases (MAPKs) (Maher, 1999; Willems-Widyastuti et al., 2013), phosphatidylinositol 3-kinase (PI3K)/Akt (Lin et al., 2011), signal transducer and activator of transcription (STAT) (Deo et al., 2002), and phospholipase (PL) Cγ (Sufen et al., 2011) (summarized in Figure 1). Correspondingly, the activation of these pathways serves to modulate diverse cell functions, including proliferation (Sulpice et al., 2002; Fernandes et al., 2004), differentiation (Klint et al., 1999; Dolivo et al., 2017), migration (Sufen et al., 2011), and apoptosis (Sahni et al., 2001).

Interestingly, beyond the direct effect of FGF2 in modulating cell function, recent evidence suggests that FGF2 can also function as an immune-modulatory factor that might play a role in immune homeostasis and dysfunction as well. In the following text, we will review FGF2 as an immunomodulatory factor in airway diseases, particularly asthma and COPD, which hopefully will provide new insight into the inflammation processes in these diseases.

## Implications of FGF2 in Airway Diseases

### The Physiological Role of FGF2 in the Lungs

FGF2 plays critical roles in all five stages of lung development: embryonic, pseudoglandular, canalicular, saccular, and alveolar. The alveolar formation in mice starts at birth and lasts for about a month; whereas in humans, it starts in the womb and lasts until the age of five or longer to mature (Burri, 2006). During the lung development process, FGF2 expression pattern is dynamic in different pulmonary cell types, indicating its differential role during the lung development stages. In mouse embryogenesis, mesoderm-derived Fgf1 and Fgf2 are involved in commiting the lung fate at the ventral foregut endoderm on embryonic day 8 (E8) (El Agha et al., 2017). An FGF2 immunolocalization study in developing rat fetal lung demonstrated that FGF2 protein localizes to AECs, the basement membrane, mesenchymal and mesothelial cells, as well as the extracellular matrix during pseudoglandular stage (Han et al., 1992). Also increasing is FGFR in AECs, suggesting that FGF2 may be involved in the control of AECs’ proliferation. Besides, FGF2 also localized to the branch points of airway buds, ASMCs, and putative VSMCs, indicating the role of FGF2 in airway system development. In contrast to early development stages, FGF2 is not detectable in airway epithelium in late fetal rat lung. This persisted at early postnatal rat lung, and the amount of FGF2 in ASMCs and many other cells in the alveolar region is also lesser (Powell et al., 1998). The physiological significance of this changing expression pattern of FGF2 is far from elucidated but could be linked to its regulation on alveolar development. For example, Yi et al. (2006) suggest that FGF2 and the FGFR1(IIIc) might contribute to the physiologic pulmonary cell apoptosis that is normally seen shortly after birth. This process may help to get rid of excess fibroblasts and epithelial cells so as to increase the gas exchange surface area. Although numerous studies have suggested the role of FGF2 in lung development, *Fgf2* knockout mice (*Fgf2^–/–^*) develop near normally, have no reported pulmonary abnormalities, and are fertile, indicating that other growth factors may compensate the function of FGF2 in lung development (Zhou et al., 1998).

Comparatively, in the mature lung, FGF2 can be expressed in AECs, ASMCs, VSMCs, ECs, and mesenchymal cell populations. Also expressed in the same cell types are the representative receptors of FGF2 (e.g., FGFR1, see below for more details (Gonzalez et al., 1996; Kranenburg et al., 2002, 2005). In addition, extracellular FGF2 has also been found in the epithelial basement membrane and pericellular matrix of ECs (Shute et al., 2004). Indeed, FGF2 is the main growth factor that is anchored to the ECM and basement membrane through preferential binding to glycosaminoglycan (GAG) side chains of cell-associated or ECM-associated heparan sulfate proteoglycans (HSPG) (Nugent and Iozzo, 2000). This interaction offers protection from proteolysis and serves as a reservoir of stable, but inactive FGF2, requiring release for biological activity (Bosse and Rola-Pleszczynski, 2008). Furthermore, its storage presumably leads to rapid cellular signaling in the face of sudden changes in local environment conditions (Redington et al., 2001). A number of mechanisms for the release of FGF2 from ECM and basement have been identified. These include proteolytic cleavage of HSPG core protein by heparinases, the action of GAG degrading enzymes, and the ability of heparin or endoglycosidase to elute FGF2 from HSPG-binding sites (Bosse and Rola-Pleszczynski, 2008). The release of FGF2 then enables it to bind to FGFRs with varying affinities on the plasma membrane.

### FGF2 Expression Pattern in Airway Diseases

**FIGURE 2:**
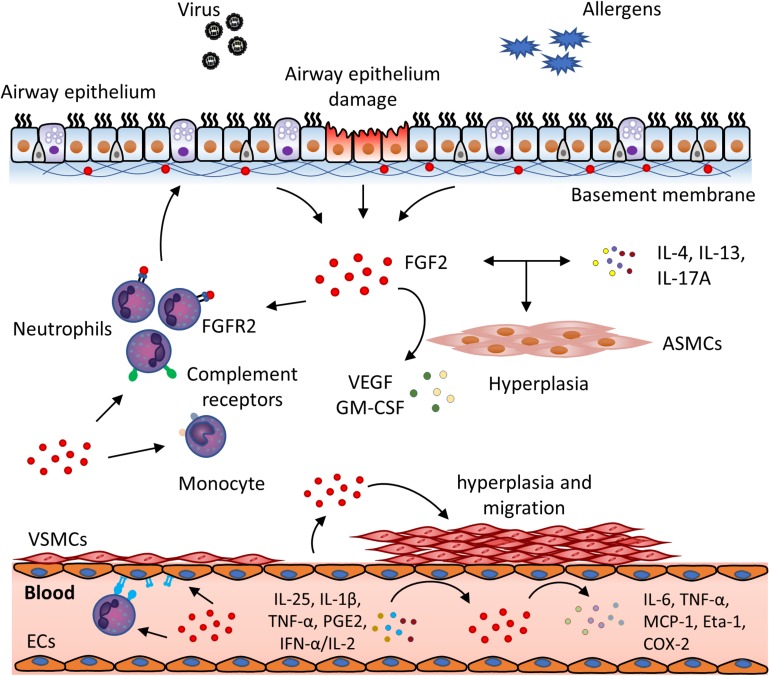
FGF2 exerts immunomodulatory function in viral infection and chronic airway inflammation. In the presence of viral infection and allergen exposure, FGF2 is secreted from damaged airway epithelial cells and released from extracellular storage. FGF2 then plays a role in the interplay of inflammatory cells and airway structural cells. In the airways, FGF2 could (1) promote neutrophils recruitment and activation through FGFR2 and complement receptors; (2) activate monocytes via upregulation of complement receptors; (3) cooperate with inflammatory cytokines, e.g., IL-4, IL-13, and IL-17A, to induce ASM hyperplasia, and (4) promote ASM to release VEGF and GMSF. In the blood vessel, excess FGF2 can be secreted by ECs in presense of inflammatory mediators, such as IL-25, IL-1β, TNF-α, PGE2 and IFN-α/IL-2. FGF2 could then (1) upregulate the expression of adhesion molecules on ECs and neutrophils, which promotes neutrophil infiltration and transmigration; (2) further activate ECs to produce inflammatory mediators, e.g., IL-6, TNF-α, MCP-1, Eta-1 and COX-2, and (3) promote VSMCs hyperplasia and migration to contribute to vascular remodeling. (ASMC, airway smooth muscle cell; COX-2, cyclooxygenase-2; EC, endothelial cell, Eta-1, osteopontin; FGF, fibroblast growth factor; FGFR, fibroblast growth factor receptor; GM-CSF, granulocyte-macrophage colony-stimulating factor; IFN, interferon; MCP-1, monocyte chemoattractant protein-1; PGE2, prostaglandin E2; TNF-α, tumor necrosis factor α; VEGF, vascular endothelial growth factor; VSMC, vascular smooth muscle cell). (Okamura et al., 1991; Cozzolino et al., 1993; Barleon et al., 1996; Wempe et al., 1997; Kage et al., 1999; Takagi et al., 2000; Ohsaka et al., 2001; Kranenburg et al., 2002; Santos et al., 2002; Bonacci et al., 2003; Lee et al., 2004; Mor et al., 2004; Shute et al., 2004; Bosse et al., 2006, 2008; Andres et al., 2009; Finetti et al., 2009; Wang et al., 2012; Ogawa et al., 2018).

There is great interest in the potential role of FGFs in the pathophysiology of chronic inflammatory airway diseases. Studies suggest that FGF2 expression is clinically relevant in these diseases. For example, FGF2 levels in the bronchoalveolar lavage fluid (BALF) of atopic asthma patients are significantly higher than healthy controls. Importantly, it can be further elevated in response to allergen exposure 10 min after segmental bronchoprovocation (Redington et al., 2001). This could be attributed to the activation of mast cells and AECs, which releases FGF2 and the desequestration of FGF2 from the ECM mediated by mast cell-produced heparin (Redington et al., 2001). Moreover, FGF2 levels in the sputum could be a biomarker for asthma severity in light of the correlation of its levels with lung function, implicating its role in airway hyperresponsiveness, the main feature of asthma (Bissonnette et al., 2014). The significance of FGF2 in disease severity also lies in COPD conditions, as FGF2 levels are elevated in the serum of patients with exacerbated COPD as compared to patients with stable COPD and healthy subjects (Pavlisa et al., 2010). In support, immunohistochemistry analysis has shown that FGF2 expression increased in the airways of asthmatic and COPD patients. For example, Kranenburg and colleagues demonstrated that the expression of FGF2 and FGFR1 were increased significantly in the bronchial epithelium in COPD patients as compared with non-COPD. Likewise, another study suggested that epithelial FGF2 was significantly more abundant in patients with mild asthma than in healthy subjects (Shute et al., 2004). Moreover, the overexpression of FGF2 in VSMCs and ECs of the small pulmonary vessels and FGFR1 in both large and small vessel types in COPD may be involved in regulating the process of pulmonary vascular remodeling (Kranenburg et al., 2002, 2005). Altogether, these data suggest that FGF2 expression is elevated in multiple pulmonary cell types in asthma and COPD, and may be involved in the pathogenesis of chronic inflammatory airway diseases. Besides its effect in tissue remodeling, there is an emerging role of FGF2 in airway inflammation, which has placed this molecule as a common link between airway inflammation and airway structural cells. Therefore, in the next section, we will review the immunomodulatory function of FGF2 in both inflammatory cells and airway structural cells (summarized in Figure 2), and discuss the possibility of targeting FGF2 so as to inhibit airway chronic inflammation in asthma and COPD.

### FGF2 in Inflammatory Cells

Asthma and COPD are chronic inflammatory airway diseases that are characterized by the infiltration of inflammatory cells. They can be recruited from circulation within minutes to hours when the body responds to stimuli, such as allergens. Interestingly, it has been shown that FGF2 can be released from those inflammatory cells including T lymphocytes, eosinophils (Hoshino et al., 2001), mast cells (Kearley et al., 2011), macrophages (Yum et al., 2011), and myeloid dendritic cells (Sozzani et al., 2007), which might partly explain the elevated FGF2 expression in the BALF of asthma patients after allergen stimulation. More importantly, elevated FGF2 modulates inflammatory cell recruitment and activation as well. In this scenario, Takagi et al. (2000) showed that FGF2 regulates the expression of adhesion molecules, i.e., L-selectin and CD11b, on the surface of blood neutrophils, the most abundant inflammatory cells present in the airway of COPD patients (Pesci et al., 1998). These adhesion molecules mediate the adherence of circulating neutrophils to vascular endothelial cells and thus facilitate the recruitment of neutrophils into the airways (Takagi et al., 2000). Moreover, two studies have demonstrated that FGF2 amplifies the inflammatory cells’ function in bacterial clearance through increasing the complement receptors (CRs), such as CR3 on blood monocytes and CR1 on neutrophils, and oxidative product formation (Takagi et al., 2000; Ohsaka et al., 2001). Interestingly, the latter is not a direct effect of FGF2, for FGF2 itself does not enhance the production of intercellular H_2_O_2_ in neutrophils, indicating that FGF2 might coordinate with other factors in modulating inflammatory cell function.

While the present evidence suggests FGF2 as an immunomodulatory factor in regulating inflammatory cell recruitment, adhesion, and cell function, unfortunately, there is not much evidence documenting the effect of FGF2 on inflammatory cells in asthma and COPD. Therefore, further studies will be required to determine the direct effect of FGF2 and, subsequently, the underlying mechanisms.

### FGF2 in Airway Structural Cells

Airway structural cells, including AECs, ASMCs, and ECs, are the central effector cell types in asthma and COPD. Beyond their effect in tissue remodeling and impairing lung function; increasing studies have focused on their role in modulating airway inflammation. This is related not only to the immunomodulatory function of these cells, but also their crosstalk with inflammatory cells that shapes the immune network. Strikingly, FGF2, and its specific receptor, FGFR1, are overexpressed in airway structural cells under diseased conditions, as mentioned above. In this context, FGFs might be the key to orchestrate the immunomodulatory function of typical airway structural cells and their crosstalk with inflammatory cells, which therefore could serve as an attractive therapeutic target for chronic airway inflammation. For the next sections, we will further discuss the role of FGF2 in different airway structural cell types, by which the role of FGF2 as an orchestrator in airway inflammation will be delineated.

#### Airway Epithelial Cells

AECs play a pivotal role in host defense against a wide range of environmental insults by acting as the first barrier. It is critical in innate immunity against pathogens and allergens, which trigger the following inflammatory processes. In some studies, this is critical for the onset and exacerbation of asthma and COPD (Gohy et al., 2015; Ladjemi et al., 2018). By interacting with AECs, FGF family members, mostly FGF7 and FGF10, are involved in innate immunity directly and indirectly (Wu et al., 2011; Gardner et al., 2016; Yuan et al., 2019). Comparatively, the role of FGF2 in AECs mediated innate immunity is also attracting research interest. For example, in Liu’s study, cytosolic FGF2 stabilizes inactivated retinoic-acid inducible gene-1 (RIG-1), the primary pathogen recognition receptor (PRR) against virus infection, via preventing proteasome-mediated RIG-1 degradation in physiological conditions. Whereas upon viral infection, FGF2 suppresses antiviral signaling by inhibiting the interaction of activated RIG-1 with downstream mitochondrial antiviral-signaling protein (MAVS) and type I interferon production (Liu et al., 2015) (Figure 1). Of note, the above-mentioned study suggests FGF2 as a negative regulator for innate immunity, and Wang et al. reported contradicting results. They showed that the increased level of secreted FGF2, which is predominately derived from AECs induced by H1N1 infection, protects recruiting, and activating neutrophils (Wang et al., 2018). This inconsistency might be due to the difference between cytosolic and secreted FGF2 protein in AECs upon viral infection.

AECs mediated innate immunity plays a crucial role in initiating allergen-induced inflammatory pathways in asthma and COPD. In addition to the function of AECs in recruiting immune cells, which subsequently link to adaptive immunity and inflammation in asthma and COPD, AECs themselves serve to be the reservoir for inflammatory cytokines in responding to stimulations. A study showed that FGF23, another FGF family member, enhances IL-1β production in primary bronchial epithelial cells from COPD patients through activating FGFR4/PLCγ/calcineurin/nuclear factor of activated T-cells (NFAT) pathway (Krick et al., 2018). Furthermore, FGF23 and TGF-β could increase the transcripts of IL-8, an essential chemokine in neutrophils recruitment (Krick et al., 2017), which further supports the role of FGF family members in epithelial-mediated inflammation. In-depth studies need to be conducted to fully elucidate the role of FGF2 in innate immunity in the different context of conditions.

Whilst FGF2 modulates AECs-mediated innate immunity and contributes to airway inflammation, not to ignore is that FGF2 might also play a role in epithelial repair and barrier function. Indeed, FGF2 has been characterized as a protective factor in repairing epithelium. As to bleomycin-induced lung injury and pulmonary fibrosis, more *Fgf2* knockout mice succumb to death due to compromised epithelial repair process and barrier function recovery (Guzy et al., 2015). This indicates that FGF2 provides a protective endogenous epithelial reparative signal in the setting of lung injury. However, the mechanism underlying the protective effects of Fgf2 signaling during airway injury has not yet been fully elucidated. Also, FGF2 may be involved in epithelial repair not only by exerting its intrinsic role but also cooperating with other inflammatory cytokines or mediators. This was supported by Song’s study that FGF2 cooperates with IL-17 to repair damaged intestinal epithelium through the Act1-mediated signaling pathway (Song et al., 2015). Nevertheless, whether this is the case in the lung remains to be elucidated.

#### Airway Smooth Muscle Cells

ASMCs are recognized as the main effector cell type in asthma and COPD. Both clinical and animal model studies suggest that the ASM layer is significantly thicker in chronic inflammatory diseases, thereby contributinge to airflow limitation and impairment of lung function. Importantly, on top of its contractile properties to induce airway constriction, there is an increasing research interest for the immunomodulatory role of that ASM played in perpetuating chronic airway inflammation (Damera and Panettieri, 2011). FGF2 has been shown to contribute to ASM hyperplasia, in light of the mitogenic property of FGF2 in ASMCs, which might play an indirect role in the pathogenesis of chronic airway inflammation. This mitogenic effect of FGF2 also lies at the interplay between FGF2 and remodeling associated molecules, typically TGF-β (Bosse et al., 2006), through the autocrine platelet-derived growth factor (PDGF) loop. In this context, FGF2 increases the expression of PDGF receptor (PDGFR), as well as tissue plasminogen activator (tPA) that is a protease known to activate PDGF ligands. This is accompanied by the increased production of PDGFR ligands (PDGF-AA and PDGF-CC) induced by TGF-β (Bosse et al., 2006). Other than this, FGF2 and TGF-β are coexpressed in the remodeled airway *in vivo*, by peribronchial mononuclear cells. In parallel, FGF2 induces TGF-β overexpression in macrophages *in vitro*, which may amplify the interaction of FGF2 and TGF-β in inflammation status (Yum et al., 2011). In addition to TGF-β, the inflammatory cytokines including IL-4, IL-13, and IL-17, the pivotal mediators driving the pathogenesis of asthma and COPD, enhance FGF2-mediated ASMCs’ proliferation in different experiment settings (Bosse et al., 2008; Ogawa et al., 2018), which further support the interaction of FGF2 with inflammation on tissue remodeling in disease status.

On the other hand, there is evidence that FGF2 might be a direct immunomodulatory factor by inducing the secretion of pro-inflammatory mediators, including growth factors, cytokines, and chemokines in ASMCs. Reported by Willems-Widyastuti, FGF2 regulates the production of vascular endothelial growth factor (VEGF) in ASMCs, which may induce T cell differentiation and monocytes recruitment (Barleon et al., 1996; Mor et al., 2004), through MAPK pathway (Willems-Widyastuti et al., 2011). In another example, FGF2 significantly increases the levels of macrophage colony-stimulating factor (GM-CSF) in ASMCs (Bonacci et al., 2003), which is critical to activate macrophages in asthma and COPD, indicating a role for FGF2 in immune cell activation. Except for this, FGF2 also plays a role in the contact-dependent communication between immune cells and ASMCs, which is featured in chronic airway diseases and being critical for ASM functional changes and immune cell survival (Ramos-Barbon et al., 2005). In this scenario, FGF2/FGFR signaling in ASMCs is thought to trigger the formation of lymphocyte-derived membrane conduits, which are a continuum of cell membrane extensions and connect ASMCs and activated CD4^+^ T cells (Farahnak et al., 2017).

#### Endothelial Cells

Despite the limited research on pathological changes of ECs in asthma and COPD compared to AECs and ASMCs; ECs have proven to be an indispensable participant in shaping the immune and inflammatory network in airway diseases (Asosingh et al., 2018). ECs play critical roles in regulating vessel tone, cellular adhesion, thromboresistance, smooth muscle cell proliferation, and vessel wall inflammation through production of multiple factors in response to different stimulis. In asthma and COPD, endothelial dysfunction, along with vessel inflammation, has been observed (Green and Turner, 2017). Moreover, in asthmatic children, the extent of endothelial dysfunction, as manifested by excess production of circulating vascular cell adhesion molecule-1 (sVCAM-I), was found to be associated with asthma severity (Butov et al., 2019), indicating the critical role of ECs in asthma pathogenesis. Indeed, in light that ECs serves as the gateway of inflammatory cells’ transendothelial migration into the lung parenchyma, the activation of ECs, e.g., expression of adhesion molecules and pro-inflammatory mediators, would be crucial in modulating inflammatory cell recruitment. In support of this, Oelsner et al. (2013) demonstrated that the expression levels of adhesion molecules on the surface of ECs are inversely related to lung function. In another study, the eotaxin mRNA was shown to be increased in ECs from asthma patients, and the level was associated with airway hyperresponsiveness (Ying et al., 1997), which further supports the role of ECs in modulating airway inflammation in chronic inflammatory diseases such as asthma and COPD.

ECs are key immunomodulatory cells in airway diseases, but the involvement of FGF2 in the dysfunction of ECs in asthma and COPD patients has not been well studied yet. However, FGF2 has been proven as an effective regulator of ECs-mediated angiogenesis and inflammation in different experimental settings. It has been well established as a potent inducer of angiogenesis that exert its effect on EC proliferation (Sahni and Francis, 2004), differentiation (Klint et al., 1999), and migration (Pintucci et al., 2002), directly or indirectly by inducing other angiogenetic factors, such as Heparin-binding EGF-like growth factor (HBEGF) and platelet-derived growth factor, B polypeptide (PDGFB) (Andres et al., 2009). FGF2 production and release from ECs can be triggered by inflammatory mediators, e.g., IL-25, IL-1β, TNF-α, prostaglandin E2 (PGE2) and IFN-α/IL-2 (Okamura et al., 1991; Cozzolino et al., 1993; Lee et al., 2004; Finetti et al., 2009; Wang et al., 2012), as well as cell damage and hypoxia (Pintucci et al., 1999; Luo et al., 2011). These findings suggest that inflammation might cooperate with FGF2 to orchestrate the amplification loop of the angiogenic response in ECs. Of note, there are also negative feedback mechanisms that contradict the angiogenetic effect of FGF2. Such examples are C-X-C Motif Chemokine Ligand 13 (CXCL13), CXCL4/CXCL4L1, and long-pentraxin 3 (PTX3) (Spinetti et al., 2001; Rusnati et al., 2004; Struyf et al., 2004; Presta et al., 2018), which need to be carefully evaluated in different diseases and inflammation types.

In addition to its angiogenetic effect, FGF2 may amplify the EC-mediated inflammation. According to recent *in vitro* studies, FGF2 may stimulate the production of various pro-inflammatory factors and chemoattractants, including IL-6, TNF-α, and monocyte chemoattractant protein 1 (MCP-1) in ECs (Wempe et al., 1997; Andres et al., 2009). These factors are essential for immune cell proliferation, survival, activation, and trafficking (Wempe et al., 1997; Li et al., 2018; Mehta et al., 2018). In another two studies, FGF2 could also induce the expression of extracellular matrix protein osteopontin (OPN/Eta-1) and cyclooxygenase-2 (COX-2) in ECs. The former is an important component of cellular immunity and inflammation (Leali et al., 2003), whereas the latter is responsible for the production of prostanoids, a key factor associated with the pathological processes of inflammatory diseases (Kage et al., 1999). Furthermore, FGF2 itself is involved in inflammatory response as well. In Okamura’s study, FGF2 inhibition completely blocked TNF-α-induced IL-6 production (Okamura et al., 1991). This suggests to us that FGF2 could be critical in inflammatory processes, and thus might be a key modulator in chronic inflammatory diseases, such as asthma and COPD.

Finally, FGF2 potentiates inflammatory cell recruitment by enhancing EC surface adhesion molecules expression. FGF2 increased the expression of ICAM-1, VCAM-1, *E*-selectin, and *P*-selectin on ECs, resulting in more efficient leukocyte migration into the surrounding tissues in an acute dermal inflammation model (Zittermann and Issekutz, 2006). Interestingly, in another aspect, FGF signaling is critical to maintaining vascular integrity and endothelial barrier function (Gillis et al., 1999; Komarova and Malik, 2008; Murakami et al., 2008; Hatanaka et al., 2012). In this regard, the recruitment of inflammatory cells induced by FGF2 may be independent of increasing vascular permeability, but rather the induction of adhesion molecules, as stated above.

Of note, whilst numerous studies support the pro-inflammatory effect of FGF2 in ECs, the anti-inflammatory activity of FGF2 has also been observed. For example, by pretreating human umbilical vein ECs with recombinant FGF2 protein for 3 days, the reduced leukocyte adhesion and transendothelial migration induced by stimulation can be observed (Griffioen et al., 1996). Further studies will be required to determine how FGF2 exert its immunomodulatory effect in chronic inflamed diseases.

#### Other Cell Types

In addition to AECs, ASMCs, and ECs, other pulmonary cells also exhibit pathological changes in asthma and COPD. Examples of these cell types include VSMCs and fibroblasts, which are critical components of vascular and airway remodeling as a result of dysregulated cell proliferation, differentiation, activation, and migration (Kranenburg et al., 2002; Santos et al., 2002). It is noteworthy that these cell types may also play active roles in chronic inflammation (Kendall and Feghali-Bostwick, 2014; Stenmark et al., 2018). In consideration of the potential of FGF2 to function as an immunomodulatory factor in AECs, ASMCs, and ECs, FGF2 may play also play similar roles in VSMCs and fibroblasts as well. In support of this, evidence showed that FGF2 is overexpressed in VSMCs from COPD patients; whereas in another study, FGF2 promotes a proinflammatory phenotype of VSMCs through increasing cellular IL-1α secretion (Schultz et al., 2007). As for fibroblast, FGF2 was shown to be a potent pulmonary fibroblast mitogen *in vitro* (Khalil et al., 2005). Moreover, FGF2 was also shown to upregulate the transcription of S100A8, a marker of inflammation, in murine fibroblast, through the MAPK pathway (Rahimi et al., 2005). These studies thus warrant future investigations into FGF2’s role in these cells.

## FGF2 as a Potential Therapeutic Target in Asthma and COPD

As outlined in the previous sections, uncontrolled airway inflammation is shown to be the key driving force for the pathogenesis of asthma and COPD. Therefore, much effort has been devoted to the development of anti-inflammatory drugs to control the disease. These drugs include both broad-spectrum anti-inflammatory medicine, typically corticosteroids, and more recently targeted drugs such as anti-IL5 and anti-IL13 were added to the repertoire of treatments. However, due to the heterogeneity of asthma and COPD, these drugs are often not applicable to certain phenotypes and endotypes in some group of patients (Brightling et al., 2015; Narendra and Hanania, 2019). Additionally, these drugs are also not targeting the crosstalk between inflammation and airway structural cells, which has proven to be the critical component in the network of sustained airway inflammation. In this scenario, we highlighted FGF2 as an important immunomodulatory factor that mediates the interplay between inflammation and airway structural cells and emphasize FGF2 as a potential therapeutic target for asthma and COPD.

Targeting FGF2 and their receptors has already been done in the drug development for cancer and other diseases, where they can potentially be applied to asthma and COPD. These drugs include chemicals to suppress FGF2 expression levels, anti-FGF2 antibody, FGFR inhibitors and recombinant FGF2 (Fu et al., 1998; De Aguiar et al., 2016; Lv et al., 2018; Loriot et al., 2019). Some drugs have been approved by the FDA, while others are in clinical trials and have been shown to have mild side effects. Encouragingly, the application of FGF2 targeted therapy has recently been expanded to pulmonary diseases. For example, neotuberostemonine, a natural alkaloid isolated from *Stemona tuberosa*, was applied to decrease the levels of FGF2 in bleomycin-induced pulmonary fibrosis mice (Lv et al., 2018). In another early report, colchicine inhibited FGF release from alveolar macrophages *in vitro* (Peters et al., 1993). These reports hence highlighted the feasibility of targeting FGF2 in asthma and COPD. Nevertheless, prior to developing these drugs for clinical use, extensive research is needed to study the role of FGF2 in different cell types and disease states. For instance, FGF2 may function as an inflammatory enhancer by regulating the functions of inflammatory cells and airway structural cells. Conversely, FGF2 may also serve protective effects on minimizing lung injury and maintaining tissue integrity. The latter was supported in a study of bleomycin-induced lung injury model (Guzy et al., 2015). It is also interesting to note that there were two studies of *in vivo* mouse models of acute asthma and COPD which showed that recombinant FGF2 (rFGF2) is protective against airway inflammation and lung function, rather than enhancing inflammation (Jeon et al., 2007; Kim et al., 2018). The inconsistencies between *in vivo* and *in vitro* studies might be due to the nature of different experiment setups, as well as the limitations of *in vivo* and *in vitro* models in reproducing clinical characteristics. For example, the mouse model of asthma used was acute inflammation model in which chronic airway inflammation and remodeling is usually absent. This might lead to different inflammatory components, wherein airway remodeling and angiogenesis are not involved. In such acute models, FGF2 treatment might help to repair acute lung injury and maintain tissue integrity, and thereby is protective by counteracting acute inflammation-induced tissue damage and increased permeability (Guzy et al., 2015). In contrast, in chronic inflammation, FGF2 might tend toward an inflammatory amplifier via interaction with inflammatory cells and airway structural cells. Moreover, rFGF2 treatment may exacerbate angiogenesis, as demonstrated in the clinical usage of FGF2 in chronic coronary artery disease and critical limb ischemia (Udelson et al., 2000; Ono et al., 2018). Therefore, more clinical studies and chronic disease models are required to fully elucidate the immunomodulatory and repair functions of FGF2 in different experimental settings prior to its clinical application.

## Future Directions and Conclusion

In this review, we provide a comprehensive analysis of FGF2’s function as an immunomodulatory factor in chronic airway diseases, with emphasis on asthma and COPD. Nevertheless, questions remain to be addressed regarding the effect of FGF2 in pulmonary cell types and diverse disease endotypes. Therefore, future studies need to be conducted. These could include (1) FGF2 expression patterns in the presence of acute and chronic inflammation status; (2) the interaction of FGF2 with typical immune cell types in asthma and COPD; (3) role of FGF2 in modulating the function of airway structural cells in airway remodeling, inflammation, and lung function; (4) role of FGF2 in *in vivo* disease models with acute or chronic inflammation; and (5) clinical studies in asthma and COPD patients with varying disease severity. With these studies being performed, a better understanding of FGF2 biology and its immunomodulatory role in asthma and COPD could provide potential alternative options for patients that are unresponsive to current anti-inflammatory treatments.

## Author Contributions

YY and D-YW conceived of this review. YT, YQ, and YY drafted this manuscript. ZC, JL, and YG designed the figures. TT and KT provided critical feedback.

## Conflict of Interest

The authors declare that the research was conducted in the absence of any commercial or financial relationships that could be construed as a potential conflict of interest.
